# Evaluation of the mechanistic basis for the antibacterial activity of ursolic acid against *Staphylococcus aureus*

**DOI:** 10.3389/fmicb.2024.1389242

**Published:** 2024-05-17

**Authors:** Guanhui Liu, Peng Qin, Xinying Cheng, Lifei Wu, Wentao Zhao, Wei Gao

**Affiliations:** ^1^School of Life Sciences and Food Engineering, Hebei University of Engineering, Handan, China; ^2^Chenguang Biotechnology Group Handan Co., Ltd., Handan, China; ^3^Hebei Plant Extraction Innovation Center Co., Ltd., Handan, China; ^4^Hebei Province Plant Source Animal Health Products Technology Innovation Center, Handan, China

**Keywords:** *Staphylococcus aureus*, ursolic acid, *Rosmarinus officinalis* L., dairy mastitis, antibacterial activity, synergistic effect

## Abstract

The antibiotics are generally regarded as the first choice approach to treat dairy mastitis, targeting the public health problems associated with the food safety and the emergence of antibioticresistant bacteria. The objective of the study was to evaluate the antibacterial efficacy of ursolic acid (UA) when used to treat *Staphylococcus aureus* and other isolates associated with bovine mastitis and to clarify the mechanistic basis for these effects. The bacteriostatic properties of UA extracted from *Rosmarinus officinalis* L. at four different purity levels were assessed by calculating minimum inhibitory concentration (MIC) values, while the synergistic effects of combining 98% UA with antibiotics were evaluated by measuring the fractional inhibitory concentration index (FICI). Changes in biofilm formation and the growth curves of the clinical isolates were assessed to clarify the bacteriostatic effect of UA. Furthermore, the cell wall integrity, protein synthesis, and reactive oxygen species (ROS) production were assessed to determine the antibacterial mechanism of UA treatment. Ultimately, UA was revealed to exhibit robust activity against Gram-positive bacteria including *S. aureus* (ATCC 25923), *Streptococcus dysgalactiae* (ATCC27957), *Streptococcus agalactiae* (ATCC13813), *Enterococcus faecalis* (ATCC29212), and *Streptococcus mutans* (ATCC25175). However, it did not affect *Escherichia coli* (ATCC 25922). The MIC values of UA preparations that were 98, 50, 30, and 10% pure against *S. aureus* were 39, 312, 625, and 625 μg/mL, respectively, whereas the corresponding MIC for *E. coli* was >5,000 μg/mL. The minimum bactericidal concentrations of 98% UA when used to treat three clinical *S. aureus* isolates (S4, S5, and S6) were 78, 78, and 156 μg/mL, respectively. Levels of biofilm formation for clinical *S. aureus* isolates decreased with increasing 98% UA concentrations. Above the MIC dose, UA treatment resulted in the dissolution of bacterial cell walls and membranes, with cells becoming irregularly shaped and exhibiting markedly impaired intracellular protein synthesis. *S. aureus* treated with 98% UA was able to rapidly promote intracellular ROS biogenesis. Together, these data highlight the promising utility of UA as a compound that can be used together with other antibiotics for the treatment of infections caused by *S. aureus*.

## 1 Introduction

Dairy mastitis is currently the most important challenge facing the dairy industry in various countries throughout the world. This reduces milk production and thereby adversely affects animal well-being while also exacting an economic toll on the dairy industry (Khan et al., 2019). Subclinical mastitis is most prevalent in North America and Uganda, while clinical mastitis is most common in the United Kingdom (Krishnamoorthy et al., 2021). Dairy mastitis is a form of intramammary inflammation that is most often caused by certain pathogenic bacterial species including *Escherichia coli*, *Streptococcus uberis*, *Staphylococcus aureus*, and *Klebsiella pneumonia* (Bar et al., 2008; Klaas and Zadoks, 2018; Machado and Bicalho, 2018). While antibiotics are generally regarded as the first choice approach to treating this devastating disease, the excessive use of antibiotics can result in the presence of residual antibiotics in foods from these animals, thus contributing to the emergence of antibiotic-resistant bacteria (ARBs). These ARBs can move from the food chain into humans and the environment, with multidrug-resistant bacteria causing an estimated 700,000 deaths per year worldwide (Krömker and Leimbach, 2017). Without appropriate measures to curb antibiotic use, it is estimated that global antimicrobial consumption in the context of the production of food animals will rise from 63,151 tons in 2010 to 105,596 tons as of 2030 (Van Boeckel et al., 2015).

These factors underscore the urgent need to explore alternatives to traditional antibiotics for use in the post-antibiotic era. Plant-derived compounds such as polyphenols (Manso et al., 2021), flavonoids (Biharee et al., 2020), terpenoids (Sycz et al., 2022), plant essential oils (Lopes et al., 2020), and alkaloids (Othman et al., 2019) have all been reported to exhibit antimicrobial activity. The natural plant derivative ursolic acid (UA, C_30_H_48_O_3_) is present in the stem bark, leaves, and peels of many Chinese herbs and fruits. UA exhibits diverse antioxidant, anti-inflammatory, antimicrobial, antitumor, hepatoprotective, and other activities (Hussain et al., 2017; Liu et al., 2023). UA or derivatives prepared therefrom exhibit robust antibacterial efficacy against Gram-positive microbes including *Streptococcus mutans* (Park et al., 2015), *S. aureus* (ATCC 6538), and methicillin-resistant *S. aureus* (MRSA) (Kefi et al., 2023; Du et al., 2024). Gram-negative bacteria are surrounded by a phospholipid-and LPS-rich outer membrane that restricts UA entry into these cells such that minimum inhibitory concentration (MIC) values for UA when used to treat Gram-negative bacteria tend to be higher than those for Gram-positive bacteria (Sycz et al., 2022).

The UA has also been reported to exhibit synergistic efficacy when applied in combination with *β*-lactam antibiotics such as ampicillin and benzacillin, enhancing the antibiotic sensitivity of *S. aureus* and *Staphylococcus epidermidis* (Kurek et al., 2012). UA (32 μg/mL) also reportedly synergizes with colistin when used to treat clinical *Klebsiella pneumoniae BC936* and *E. coli U3790* isolates (Sundaramoorthy et al., 2019). Despite the promising *in vitro* bacteriostatic activity of UA, it is not commonly used in the context of poultry or livestock production. The utilization of UA as a feed additive for animal husbandry warrants consideration as an alternative to antibiotic administration.

The antibacterial actions of UA are reported to be associated with morphological changes in the bacterial cells, inhibition of biofilm formation, and impairment of the bacterial cell membrane (Sycz et al., 2022). UA reduces biofilm synthesis by *S. mutans* by competitive inhibition of glucosyltransferases and reducing the expression of the *gft* gene that inhibits the binding of extracellular polymeric substance (EPS) in the biofilm (Liu et al., 2021; Lyu et al., 2021). According to Ren et al. (2005), UA isolated from *Diospyros dendo* leaves reduces biofilm formation by *E. coli* (including ATCC 25404), which contributes to the overexpression of *motAB* genes. UA damages the membrane integrity of MRSA and increases the concentration of Alkyl hydroperoxide reductase subunit C (AhpC) that related to the oxidative response (Wang et al., 2016).

The present study details the antibacterial activity of UA preparations of different purity levels (98, 50, 30, and 10% pure) and the synergistic effects of combining UA and antibiotics when treating *S. aureus* strains. Scanning and transmission electron microscopy (SEM and TEM) analyses were also performed, as were flow cytometry and *S. aureus* biofilm formation assays, thereby enabling the detailed examination of the mechanistic basis for the antimicrobial effects of UA. Together, these results will provide a valuable foundation for efforts to apply UA as an alternative to antibiotics when seeking to treat dairy mastitis.

## 2 Materials and methods

### 2.1 Test strains

The *Staphylococcus aureus* (ATCC25923), *Streptococcus dysgalactiae* (ATCC27957), *Streptococcus agalactiae* (ATCC13813), *Enterococcus faecalis* (ATCC29212), *Streptococcus mutans* (ATCC25175), and *Escherichia coli* (ATCC 25922) strains used in this study were obtained from Ning Bo Testobio Co., Ltd. Clinical isolates including 5 strains of *Salmonella*, 1 strain each of *Klebsiella pneumoniae* and *Fecal coliform*, 9 strains of *S. aureus* were obtained from the Innovation Center of Poultry Disease Technology of Hebei Province. The NGS sequences for the *S. aureus* S4, S5, and S6 isolates were submitted to the NCBI database (BioSample accession numbers, SAMN38649273, SAMN38673743, and SAMN38673744).

All strains were streaked for inoculation in Mueller Hinton Agar (MHA) followed by subculturing at 37°C. Individual bacterial colonies were then transferred into Mueller-Hinton Broth (MHB) and cultivated in an incubator for 24 h.

UA extracted from *Rosmarinus officinalis* L. using methanol at purity levels of 98, 50, 30, and 10% was dissolved in 12.5% (v/v) dimethyl sulfoxide (DMSO) at 10 mg/mL for experimental use. Isochlorogenic acid (diCQA) extracted from *Stevia rebaudiana* using methanol at purity levels of 60% was dissolved in ddH_2_O at 10 mg/mL for experimental use.

### 2.2 MIC and minimum bactericidal concentration (MBC) measurements

The MIC values for UA against different bacterial strains were computed using a microbroth two-fold dilution approach as per the guidelines of the Clinical and Laboratory Standards Institute (CLSI-2022) (Andrews, 2001). Initially, 100 μL of MHB medium was transferred into a 96-well plate, after which 100 μL of stock UA (10 mg/mL) was added to the wells in the first row of this plate to yield a final 5 mg/mL concentration. Then, serial two-fold dilutions of this top concentration were performed across the plate to generate a serial dilution range from 0.0024–5 mg/mL. Then, 100 μL of bacterial suspensions (1.0 × 10^6^ CFU/mL) were added to individual wells. For positive and negative control conditions, the UA stock solution or test bacteria were replaced with an antibiotic solution (ampicillin, ceftiofur hydrochloride, and tetracycline) or MHB medium. After preparation, these plates were incubated at 37°C for 16–18 h.

**Figure 1:**
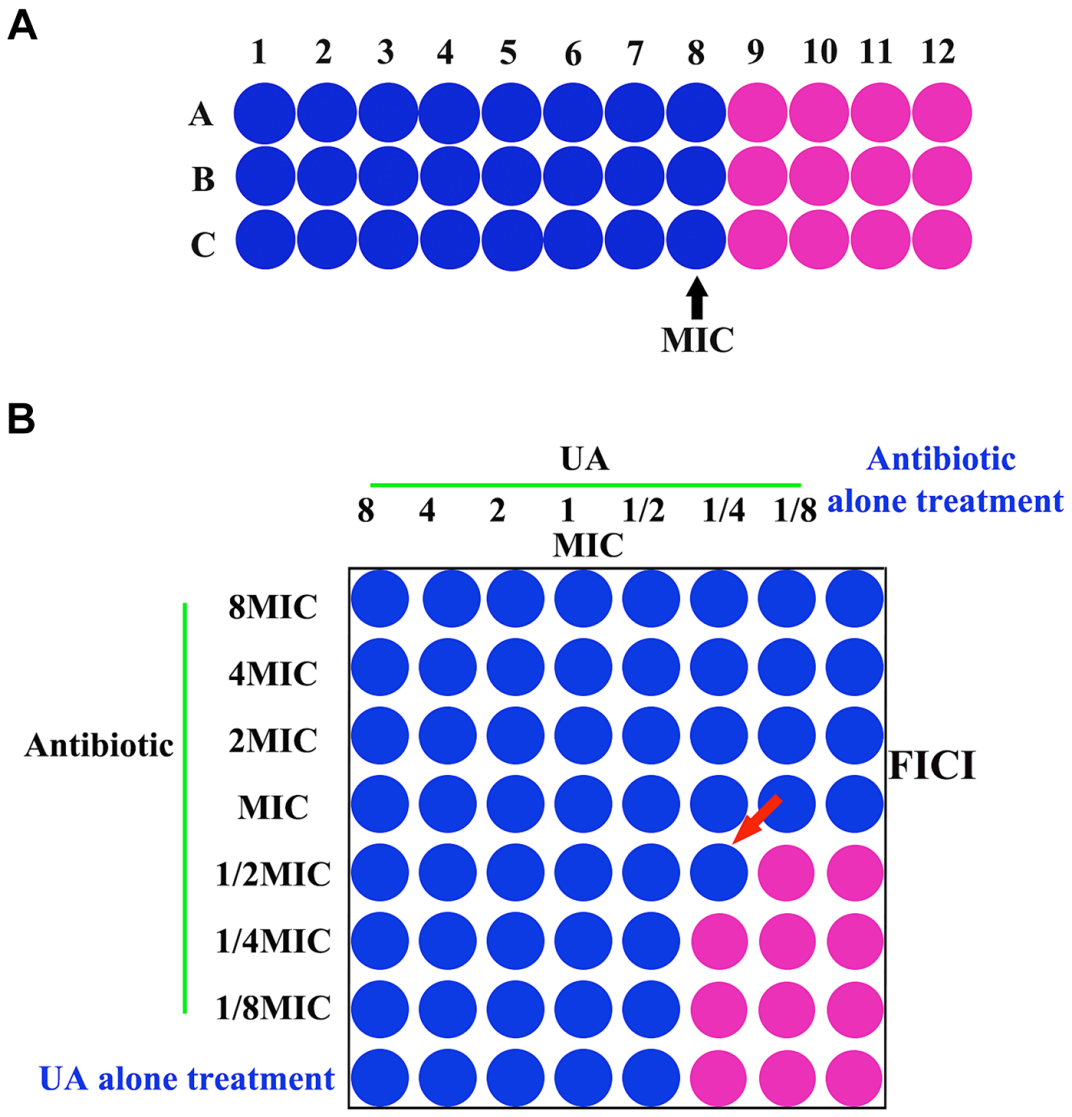
**(A)** MIC and **(B)** FICI analyses. MIC, minimum inhibitory concentration; FICI, fractional inhibitory concentration index.

The MIC values for UA were subsequently visualized using resazurin staining as a means of detecting the growth of bacteria (Caso Coelho et al., 2021). Briefly, cells were incubated for 1 h after adding 20 μL of resazurin (0.01%) per well. MIC values were identified based on the lowest UA concentration for which the wells did not turn pink in color (Figure 1A).

When determining MBC values, 100 μL of bacterial culture from the wells of concentrations exceeding the MHC value were plated on MHA and incubated for 48 h at 37°C, after which bacterial growth was assessed. The MBC concentration was identified as the lowest concentration of UA for which bacterial colonies were not observed (Wang et al., 2020).

### 2.3 Checkerboard dilution assay

Synergistic effects between UA and specific antibiotics were evaluated via a checkerboard dilution assay (Sundaramoorthy et al., 2019). Tested UA concentrations ranged from 312 μg/mL (8x MIC) to 4.8 μg/mL (1/8x MIC), while concentrations of antibiotics (ampicillin or ceftiofur hydrochloride) ranged from 15.6 μg/mL (8x MIC) to 0.24 μg/mL (1/8x MIC) μg/mL. To each well of a 96-well plate, serial dilutions of UA (50 μL) and antibiotics (50 μL) were added, after which bacterial cultures (50 μL) were added. Plates were then incubated for 24 h, after which combinatorial MICs were identified via resazurin red staining as above based on the concentrations for which no visible bacterial growth was evident (Figure 1B). Fractional inhibitory concentration index (FICI) values were computed as a means of assessing interactions between UA and antibiotics as follows: FICI = FICA + FICB = [A]/MICA + [B]/MICB, where [A] and [B] respectively denote the UA and antibiotic concentrations, while MICA/FICA and MICB/FICB correspond to the respective MIC/FIC values for UA and antibiotics. A FICI <0.5, 0.5–0.75, 0.75–1, 1–4, and >4, respectively, indicated synergy, partial synergy, an additive effect, no effect, and antagonism (Sundaramoorthy et al., 2019).

### 2.4 Growth curve analyses

To prepare a fresh bacterial culture, cells were grown at 37°C for 24 h. A bacterial suspension was then treated with 98% UA at concentrations ranging from 2x MIC to 1/2x MIC in two-fold serial dilutions, followed by incubation at 37°C. Negative and positive control bacterial suspensions were also prepared. Over the course of the 24 h incubation period, absorbance values at 600 nm were recorded for all wells and plotted against time to quantify the effects of UA on bacterial growth.

### 2.5 Biofilm formation assay

Bacteria were treated with 98% UA in two-fold serial dilutions based on the determined MIC values for 48 h, after which media was collected and cells were washed three times using PBS. Biofilms were then fixed for 15 min using 200 μL of methanol, allowed to dry naturally, and stained with 20 μL of 0.1% (w/v) crystal violet. The stain was then removed and plates were washed three times with 200 μL of ddH_2_O per well, after which 100 μL of 33% glacial acetic acid (v/v) was added to each well at room temperature for 10 min to dissolve samples, and absorbance at 570 nm was subsequently measured using a microplate reader.

### 2.6 SDS-PAGE analyses

*Staphylococcus aureus* protein profiles were evaluated via SDS-PAGE according to Tesařová et al. (2016). Initially, these bacteria were treated with 98% UA and 1x, 2x, 4x and 8x MIC concentrations for 18 h at 37°C. The control group was not treated with 98% UA. Cells were then centrifuged and the supernatant was discarded. Proteins in the precipitated fraction were diluted in 5x loading buffer and boiled for 10 min, after which 25 μL from each sample was loaded into an SDS-PAGE gel and separated via electrophoresis (120 min, 70 V). After Coomassie brilliant blue staining, gels were washed with distilled water, enabling the visual assessment of *S. aureus* protein profiles.

### 2.7 Scanning electron microscopy

After confirming the sensitivity of bacteria to UA treatment, the impact of treatment with UA on bacterial morphology was assessed via electron microscopy (Lyu et al., 2021). Bacteria were treated with 98% UA (final concentrations of 312, 156, 78, and 0 μg/mL) for 18 h, after which they were centrifuged and pellets were collected, fixed using 2.5% glutaraldehyde for 16 h at 4°C, washed three times using 0.1 M phosphate buffer (pH 7.0; 15 min/wash), and post-fixed for 2 h with 1% osmium tetraoxide. Cells were then washed with 0.1 M phosphate buffer (pH 7.0), dehydrated using an ethanol gradient (35–100%), treated for 30 min with a 1:1 ethanol and isoamyl acetate mixture, and then treated for 1 h using pure isoamyl acetate. The resultant cell suspension was dried, gold coated, and imaged with a Hitachi S-4800 Scanning Electron Microscope.

### 2.8 Transmission electron microscopy

Specimens were initially prepared as in section 2.7 above, after which they were initially dehydrated using an ethanol gradient (30–100%, 15 min/step) and incubated for 20 min in absolute acetone (Zhou et al., 2017). For infiltration, samples were placed into a 1:1 mixture of pure acetone and Spurr resin mixture for at room temperature for 1 h, followed by incubation for 3 h in a 1:3 mixture of pure acetone and Spurr resin mixture, and a final overnight incubation in Spurr resin mixture. For embedding and sectioning, the samples were transferred into Eppendorf tubes containing Spurr resin and heated for 9 h at 70°C, after which they were sectioned using a LEICA EM UC7 ultramicrotome and stained for 5–10 min using uranyl acetate and alkaline lead citrate, followed by TEM analysis with a Hitachi Model H-7650 instrument.

### 2.9 ROS analyses

Bacterial intracellular ROS levels were analyzed with 2′,7′-dichlorodihydrofluorescein diacetate (DCFH-DA, Beyotime, China), a fluorescent probe that can undergo deacetylation and oxidation to yield a fluorescent product (Wu et al., 2019). Following treatment with 98% UA (final concentrations of 625, 312, 156, 78, and 0 μg/mL), *S. aureus* was fixed using 4% paraformaldehyde, stained in the dark for 30 min with DCFH-DA, and promptly analyzed by flow cytometry (Guava easyCyte 12HT).

### 2.10 Statistical analysis

Experiments were independently repeated in triplicate, and the results were compared with one-way ANOVAs and Tukey’s *post hoc* test. Different letters indicate significant differences at *p* < 0.05. GraphPad Prism 8 was used to generate all figures.

## 3 Results

### 3.1 Minimum inhibitory concentration analyses

The antimicrobial potential of UA preparations of different purity levels was evaluated against a range of Gram-positive and Gram-negative bacteria (Table 1). The MIC values for UA preparations that were 98, 50, 30, and 10% pure when used to treat *S. aureus* ATCC 25923 were 39, 312, 625, and 625 μg/mL, respectively, whereas the corresponding value for Gram-negative *E. coli* was >5 mg/mL. The MIC values for diCQA when used to treat *S. aureus* ATCC 25923, *S. dysgalactiae* (ATCC27957), *S. agalactiae* (ATCC13813), *E. faecalis* (ATCC29212), *S. mutans* (ATCC25175), *E. coli* (ATCC 25922) were 2,500, 3,125, 6,250, 3,120, 6,250, and 3,125 μg/mL, respectively, thus demonstrating that UA outperformed diCQA with respect to its ability to suppress the growth of Gram-positive bacteria. Moreover, 98% UA was able to robustly inhibit the growth of 9 laboratory-adapted *S. aureus* strains, while also exhibiting weak antibacterial activity against clinical isolates including *Klebsiella pneumoniae, E. faecalis*, and *Salmonella* strains (Table 2).

**Table 1:** MIC concentrations for plant extracts and antibiotics against Gram-positive and Gram-negative ATCC bacterial strains.

Microorganisms		98% UA	50% UA	30% UA	10% UA	diCQA	Ampicillin	Ceftiofur hydrochloride	Tetracycline
		Minimal Inhibitory Concentration (μg/mL)							
Gram-positive bacterium	*Staphylococcus aureus* ATCC25923	39	312	625	625	2,500	1.95	0.975	Undetected
	*Streptococcus dysgalactiae* ATCC27957	19.5	19.5	78	78	3,125	0.048	敏感	0.39
	*Streptococcus agalactiae* ATCC13813	156	156	312	312	6,250	0.195	0.024	0.78
	*Enterococcus faecalis* ATCC29212	19.5	78	625	625	3,120	1.95	125	62.5
	*Streptococcus mutans* ATCC25175	9.75	39	78	78	6,250	0.0975	敏感	3.12
Gram-negative bacterium	*Escherichia coli* ATCC 25922	>5,000	>5,000	>5,000	>5,000	3,125	125	Undetected	Undetected

**Table 2:** MIC concentrations for different plant extracts and antibiotics against specific clinical isolates.

Microorganisms		98% UA	50% UA	30% UA	10% UA	diCQA	Ampicillin	Ceftiofur hydrochloride	Tetracycline
		Minimal Inhibitory Concentration (μg/mL)							
Gram-positive bacterium	*S. aureus* T1	9.75	39	156	156	6,250	3.9	>1,000	>1,000
	*S. aureus* T2	156	625	625	625	3,125	0.24	3.9	1.95
	*S. aureus* 1	156	312	1,250	1,250	3,125	1.95	1.95	1.95
	*S. aureus* 2	78	312	625	625	3,125	0.24	1.95	1.95
	*S. aureus* S3	625	625	625	625	3,125	125	62.5	0.975
	*S. aureus* S4	19.5	78	312	156	3,125	62.5	1.95	125
	*S. aureus* S5	19.5	78	312	312	1,560	15.6	1.95	125
	*S. aureus* S6	39	156	625	625	1,560	250	1.95	125
	*S. aureus* S7	19.5	156	625	1,250	1,560	1.95	7.8	1.95
Gram-negative bacterium	*Klebsiella pneumoniae*	>5,000	>5,000	>5,000	>5,000	25,000	>1,000	>1,000	7.8
	*Fecal coliform*	>5,000	>5,000	>5,000	>5,000	25,000	15.6	1.95	62.5
	*Salmonella* 1	>5,000	>5,000	>5,000	>5,000	25,000	>1,000	>1,000	500
	*Salmonella* 2	>5,000	>5,000	>5,000	>5,000	25,000	>1,000	>1,000	250
	*Salmonella* 3	>5,000	>5,000	>5,000	>5,000	25,000	3.9	15.6	500
	*Salmonella* 4	>5,000	>5,000	>5,000	>5,000	25,000	3.9	31.2	125

The results of FICI calculations are presented in Table 3. These data suggested that when treated with 98% UA, *S. aureus* exhibited increased susceptibility to ampicillin or Ceftiofur hydrochloride treatment. In six different clinical *S. aureus* isolates, the calculated FICI value for the AMP-UA (*S. aureus* S5) and Cef-UA (*S. aureus* T1) combinations was 0.375, indicative of synergistic interactions and consequent reductions in ampicillin or Ceftiofur hydrochloride MIC values when UA was present.

**Table 3:** FICI values for UA and antibiotics when used to treat clinical *S. aureus* isolates.

Clinical isolated *S. aureus*	S3	S4	S5	S6	S7	T1
UA	1/4	1/4	1/4	1/2	1/4	1/4
Ampicillin (MIC)	1/2	1	1/8	1/4	1	——
Ceftiofur hydrochloride	——	——	——	——	——	1/8
Fractional Inhibitory Concentration Indices (FICI)	0.75	1.24	0.375	0.75	1.25	0.375
The synergistic activities	Additive	No effect	Synergistic	Additive	No effect	Synergistic

A FICI < 0.5, 0.5–0.75, 0.75–1, 1–4, and >4, respectively, indicated synergy, partial synergy, an additive effect, no effect, and antagonism, as per the European Committee for Antimicrobial Susceptibility Testing (EUCAST) criteria.

### 3.2 Assessment of UA MBC values

Next, the MBC values for UA were calculated when used to treat three clinical *S. aureus* isolates (Table 4), revealing that the MBC values for 98% UA for the ATCC 25923, *S. aureus* S4, S5, and S6 strains were 625, 78, 78, and 156 μg/mL, respectively.

**Table 4:** MBC values for UA when used to treat clinically isolated strains.

	98% UA	50% UA	30% UA	10% UA
	MBC (μg/mL)			
*S. aureus* ATCC25923	625	1,250	1,250	1,250
*S. aureus* S4	78	625	1,250	625
*S. aureus* S5	78	156	1,250	1,250
*S. aureus* S6	156	1,250	625	625

### 3.3 Analyses of the impact of UA on *Staphylococcus aureus* growth

**Figure 2:**
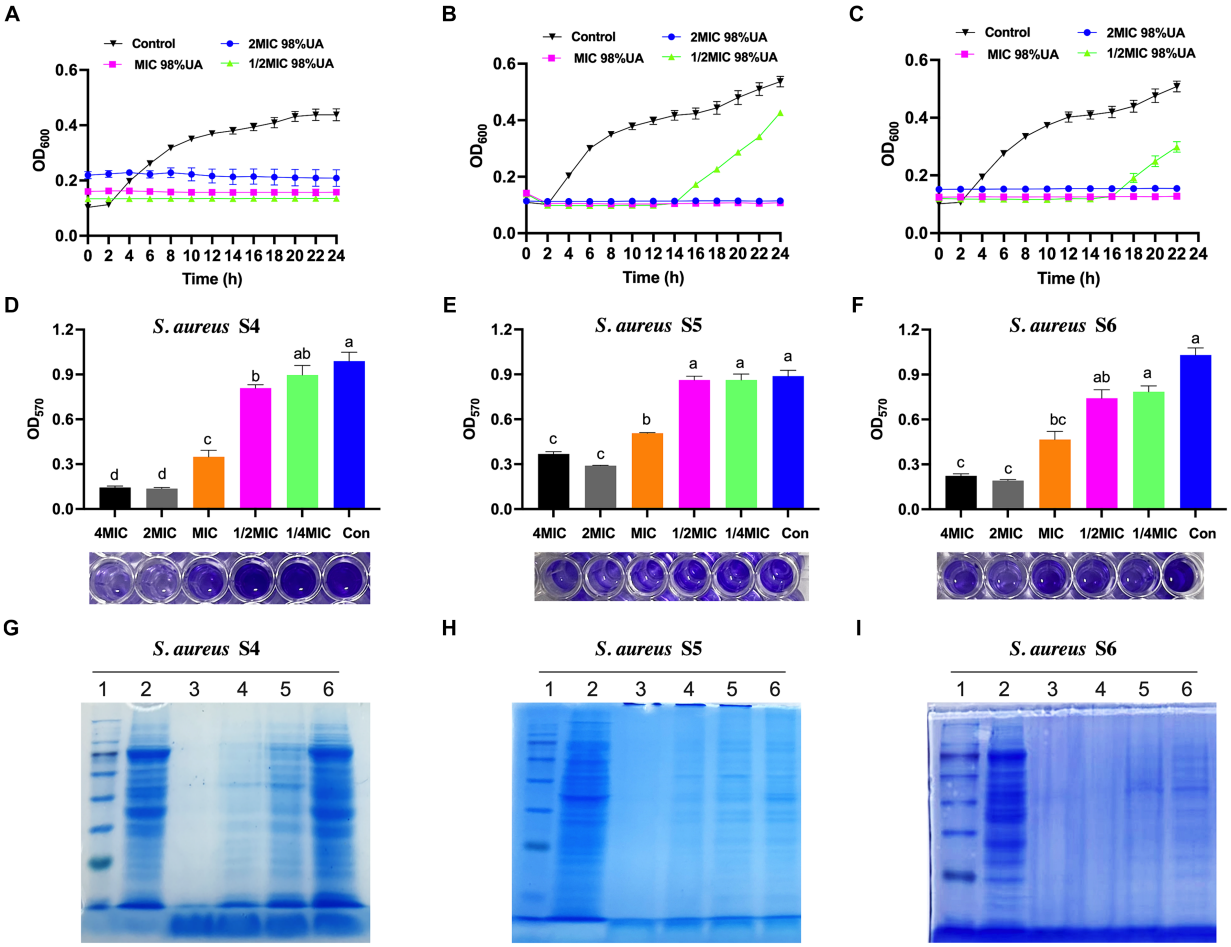
The effects of 98% UA on the growth **(A–C)**, biofilm formation activity **(D–F)**, and the bacterial protein production **(G–I)** of *S. aureus* isolates S4, S5 and S6. Con, control (untreated). Different letters indicate significant differences at *p* < 0.05. 1–6, Marker, control, 8x MIC, 4x MIC, 2x MIC, 1x MIC, respectively.

*Staphylococcus aureus* growth under conditions of 98% UA treatment was next evaluated for up to 24 h, revealing the dose-dependent inhibition of bacterial growth (Figures 2A–C). For all tested bacterial strains, treatment with a 1x MIC dose totally suppressed growth, while a 1/2x MIC concentration prolonged the bacterial lag phase. Relative to control cells, all UA-treated bacteria exhibited an earlier lag phase, a shorter log phase, and the dose-dependent suppression of stationary phase growth.

### 3.4 UA treatment partially inhibits bacterial biofilm formation

Levels of biofilm formation were next quantified via crystal violet staining, revealing that biofilm levels declined with increasing 98% UA concentration (Figures 2D–F). At the above MIC, 98% UA significantly reduced the biofilm levels by 0.65–0.86, 0.55–0.81, and 0.43–0.67-fold when comparing the control groups for *S. aureus* S4, S5, and S6, respectively (all; *p* < 0.05).

### 3.5 UA treatment suppresses bacterial protein levels

Coomassie brilliant blue staining of SDS-PAGE gels was next used to evaluate protein levels in three different *S. aureus* strains (Figures 2G–I). Relative to control cells, those cells treated with 98% UA above the MIC concentration exhibited a loss of protein bands, with the number of bacterial protein bands being negatively correlated with the 98% UA concentration.

### 3.6 UA treatment alters the morphology of *Staphylococcus aureus* cells

**Figure 3:**
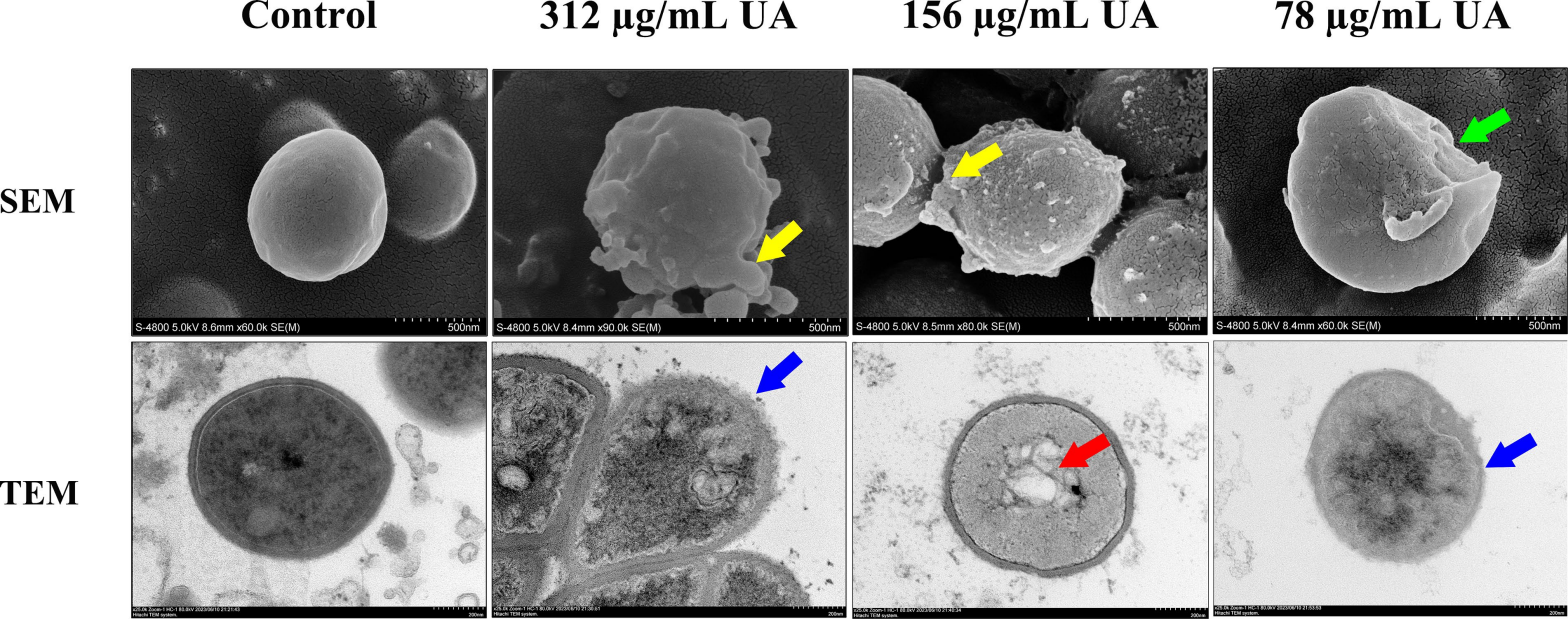
SEM and TEM analyses of *S. aureus* S5 following treatment with a range of 98% UA concentrations. Control, untreated. Yellow areas denote clear bulging of the rough *S. aureus* surface, while green arrows indicate surface sinking, blue arrows highlight the blurring of the boundary between the cell wall and membrane, and red arrows reveal the disturbance of the uniformity of the cytosol.

The effects of UA treatment on *S. aureus* morphology were next evaluated (Figure 3). Control cells were smooth, round, and exhibited clear boundaries. Following treatment with 98% UA extracted from *Rosmarinus officinalis* L. for 18 h, clear bulges or depressions in the rough surface of these *S. aureus* cells were evident, with a positive correlation between UA concentration and the number of malformed cells. The results clearly revealed that treatment with 98% UA at 312 or 156 μg/mL was associated with significant damage to the bacterial cell wall consistent with the observed antimicrobial activity of this preparation.

Ultrastructural changes in *S. aureus* are presented in Figure 3. Control cells were largely circular with an intact cell wall and a uniform cytoplasm. Following treatment with UA extracted from *Rosmarinus officinalis* L. for 14 h, the dissolution of the cell wall and membrane was evident, with cells becoming irregularly shaped and a loss of cytoplasmic uniformity. The damage to the cell wall and membrane was also associated with the blurring of the cell boundary and the release of extracellular solutes.

### 3.7 UA treatment stimulates intracellular ROS production

**Figure 4:**
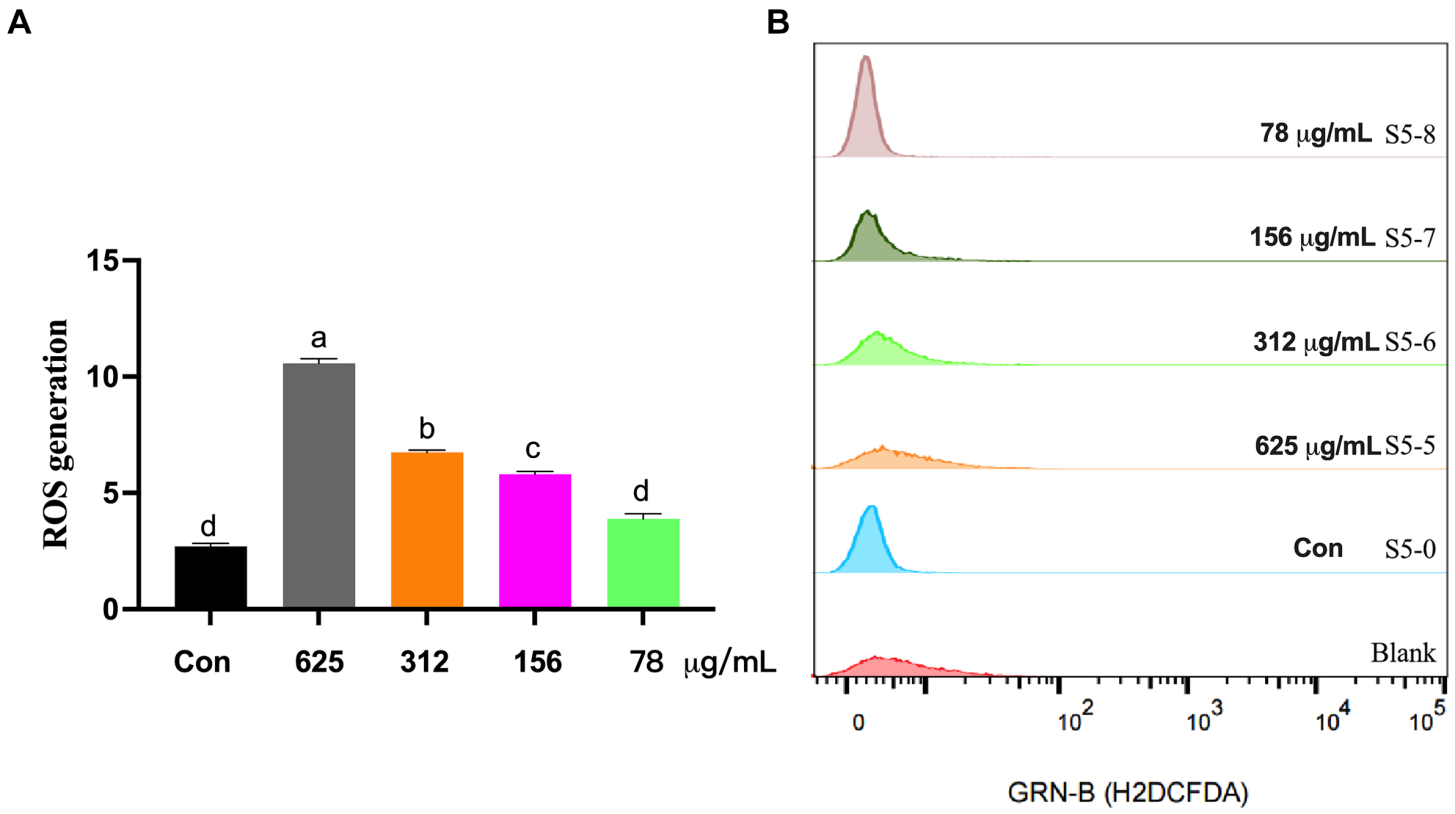
ROS levels in *S. aureus* S5 following treatment with different 98% UA concentrations. Control, untreated. Different letters indicate significant differences at *p* < 0.05. **(A)** Represents the ROS levels, and **(B)** Represents the result of H2DCFDA staining that reflect the ROS levels.

Lastly, shown in Figure 4, the levels of intracellular ROS were evaluated using the DCFH-DA probe. Relative to control cells, *S. aureus* treated with 98% UA concentrations of 625, 312, 156, and 78 μg/mL exhibited significant increases in the levels of intracellular ROS production by 291.4, 149.8, 114.9, and 43.8%, respectively, (*p* < 0.001).

## 4 Discussion

The UA has been reported to exhibit a wide range of promising uses as an antibacterial agent and antioxidant in settings including the cosmetic industry (López-Hortas et al., 2018). Sekandi et al. (2023) demonstrated that UA from *Spermacoce princeae* (K. Schum) showed effective bactericidal and antifungal activity against *C. albicans*, *S. aureus*, and *P. aeruginosa* strains (zone of inhibition diameter, 12–20 mm), while exhibiting weaker bacteriostasis against *E. coli* and *K. pneumoniae* (zone of inhibition diameter, <10 mm). Here, UA extracted from *Rosmarinus officinalis* L. (98% purity) was found to effectively inhibit the growth of *S. aureus* ATCC25923, *S. dysgalactiae* ATCC27957, *E. faecalis* ATCC29212, and *S. mutans* ATCC25175 with MIC values ranging from 9.75–39 μg/mL, while also inhibiting *S. agalactiae* ATCC13813 at a MIC of 156 μg/mL. UA was unable to inhibit the growth of *E. coli* ATCC 25922 or clinical Gram-negative isolates (*Klebsiella pneumoniae*, *Fecal coliform*, and *Salmonella*), which exhibited MIC values greater than 5,000 μg/mL. The bacteriostatic activity of UA was positively correlated with its purity in this study, and the MBC values for 98% UA ranged from 78–156 μg/mL when used to treat clinical *S. aureus* isolates. These results confirmed that UA was better able to suppress the growth of Gram-positive bacteria relative to Gram-negative bacteria, in line with prior evidence (Horiuchi et al., 2007; Wrońska et al., 2022). This is likely attributable to the fact that Gram-negative bacteria exhibit an outer membrane and express efflux pumps that prevent the intracellular entry and accumulation of UA and related compounds (Briers and Lavigne, 2015; Opperman and Nguyen, 2015). UA was able to inhibit the growth of Gram-positive *S. aureus* and *S. epidermidis* by 80% at a 20 μg/mL dose in a prior study, whereas higher concentrations (50 μg/mL) only suppressed the growth of *E. coli*, *P. hauseri*, and *C. jejuni* by 20–30% (Wrońska et al., 2022). The MIC for UA derived from the aerial portions of *Sambucus australis* against *S. aureus* (ATCC 6538) was previously measured at 32 μg/mL, whereas it exhibited moderate levels of activity against *E. coli* (ATCC 25922), *K. pneumoniae* (ATCC 10031), and *Shigella flexneri* (ATCC 12022) with a MIC of 64 μg/mL (do Nascimento et al., 2014). There is also prior evidence supporting the activity of UA extracted from *Alstonia scholaris* leaves against *E. faecalis*, *L. monocytogenes*, and *Bacillus cereus* (MIC: 1–8 μg/mL), whereas it failed to adversely affect *E. coli*, *S. enterica*, and *P. aeruginosa* (MIC >128 μg/mL) (Wang et al., 2016). UA derived from *D. melanoxylon* and *S. australis*, when used to treat a range of Gram-negative bacteria (*E. coli, K. pneumoniae*, *P. syringae*, *S. typhi*, and *S. flexneri*) with good antibacterial efficacy (MIC: 25–64 μg/mL) (Mallavadhani et al., 2004; do Nascimento et al., 2014).

In an effort to enhance the sensitivity of Gram-negative bacteria to UA, derivatives thereof with altered structural properties can be used, or UA can be applied in a synergistic manner with antibiotics (Cunha et al., 2010; Mlala et al., 2019; Wu et al., 2021; Pereira et al., 2022). For example, the MIC of a UA derivative in which the C-3 OH group had been modified to produce 3β-acetoxy-urs-12-en-28-oic acid was lower than that of unmodified UA when used to treat *S. flexneri* (ATCC 12022), *Vibrio cholerae* (ATCC 15748), *Listeria monocytogenes* (ATCC 19117), and *E. coli* (ATCC 25922). Moreover, the combination of UA and kanamycin increased *E. coli* (ATCC 25922) susceptibility, reducing MIC values from 128 to 16 μg/mL (do Nascimento et al., 2014). Here, the administration of 1/4x MIC UA plus 1/2x MIC ceftiofur hydrochloride or 1/8x MIC ampicillin was sufficient to suppress the growth of clinical *S. aureus* isolates more readily than either UA or antibiotics alone. The 98% pure UA extracted from *Rosmarinus officinalis* L. and these tested antibiotics thus exhibited good synergistic activity against dairy cow-derived *S. aureus* isolates.

*Staphylococcus aureus* demonstrated a high degree of susceptibility to UA treatment, with MIC values at the lowest tested concentrations of UA (9.75–78 μg/mL) and MBC values ranging from 9.75–156 μg/mL. The bacterial growth of three *S. aureus* strains was fully inhibited by treatment with 1x and 2x MIC doses of 98% UA, and even those bacteria treated with a 1/2x MIC dose of UA exhibited an extended lag phase followed by a less pronounced exponential growth phase from 14 to 24 h, with this prolongation of the lag phase being attributable to the inhibition of DNA replication (Rolfe et al., 2012), consistent with the ability of UA to delay bacterial growth. Biofilm formation by bacteria growth on abiotic and biotic sources is a complex process that entails adhesion, the production of extracellular polymeric substances, the formation of matrix and microcolonies, and dispersal (Rabin et al., 2015). The ability of bacteria to establish biofilms is closely related to the resistance of these microbes. Here, UA concentrations above the MIC were associated with reduced biofilm formation, with no differences in biofilm levels between the control group and *S. aureus* S5 and S6 treated with a 1/2x, 1/4x MIC dose of UA. Zhou et al. (2013) previously showed that UA from Sigma-Aldrich was able to inhibit *S. mutans* and *S. gordonii* biofilm formation at a sub-inhibitory 1/4x MIC concentration (64 μg/mL), while UA extracted from *Arctium lappa* leaves can reportedly significantly suppress *P. aeruginosa* biofilm formation at a 500 μg/mL dose (Lou et al., 2015).

Electron microscopy examinations demonstrated that UA extracted from *Rosmarinus officinalis* L. significantly altered the shapes of treated *S. aureus* cells such that they became irregularly shaped with a rough surface, in contrast to the smooth, rounded appearance of control cells. Cell wall and membrane damage were evident in treated cells, with cytoplasmic irregularities. This is similar to what has been reported previously with respect to the morphological changes evident in *S. aureus* cells treated with vine tea extract and its active ingredient, 2R, 3R-Dihydromyricetin (Liang et al., 2020). These compounds were shown to be capable of disrupting membrane permeability via increasing extracellular *β*-galactosidase content and decreasing total protein levels by 15.5 and 9.9%, respectively (Liang et al., 2020). UA treatment has also been demonstrated to suppress the viability of carbapenem-resistant *Klebsiella pneumoniae* (CRKP), inhibiting biofilm formation and inducing the downregulation of genes associated with biofilm production (*pgaA*, *luxS*, *wbbM*, and *wzm*) while also disrupting the membranes of these cells such that cytoplasmic contents were able to leak out and the overall size of the cytosol was reduced (Qian et al., 2020). These results align well with the observed electron microscopy findings for UA-treated bacteria cells, which exhibited clear evidence of cell wall damage and the leakage of intracellular contents. Therefore, the numbers of *S. aureus* proteins were decreased by UA treatment in this study detected by SDS-PAGE. Using two-dimensional (2D) proteomic analysis, it demonstrated that UA damaged the membrane integrity of MRSA, and induced the proteins involved in the bacterial phosphoenolpyruvate sugar phosphotransferase system and the oxidative response (Wang et al., 2016).

The present data support the ability of UA to suppress biofilm-related activity in *S. aureus* as previously reported by Jyothi et al. (2018). The triterpenoid madecassic acid has been shown to disrupt cell wall and cytoplasmic membrane integrity to trigger the release of contents from the cytoplasm, while also inhibiting TCA cycle activity, decreasing the activity of malate dehydrogenase and succinate dehydrogenase, and separating DNA base pairs (Wei et al., 2023). These compounds may also promote hydrophobic phenolic group accumulation within the lipid bilayer, thereby mediating lipid-protein interactions that increase the overall permeability of the membrane and destroy its integrity (Zore et al., 2011; Wu et al., 2016; Park et al., 2018). UA can impact many different genes involved in key metabolic processes in *S. mutans*, inhibiting glycolysis, fatty acid synthesis, amino acid synthesis, and peptidoglycan synthesis, thus mediating antimicrobial effects (Park et al., 2018).

It has been reported that the cell membrane integrity is crucial to prevent the excessive accumulation and production of reactive oxygen species (Wang et al., 2016; Yang et al., 2023). Prior studies have highlighted a correlation between increased ROS biogenesis and bacterial apoptosis (Li et al., 2021). Isobavachalcone flavonoids, when used to treat *S. aureus* at a 2x MIC dose, can reduce the ΔpH aspect of the proton motive force, interfering with bacterial membrane homeostasis such that ROS accumulation occurs (Song et al., 2021). To further clarify the mechanisms whereby UA kills bacterial, intracellular ROS content was assessed with DCFH-DA, revealing that 98% UA treatment at doses of 78–625 μg/mL was sufficient to significantly increase ROS levels within *S. aureus* cells by 291.4, 149.8, 114.9, and 43.8%, with a negative correlation between 98% UA concentrations and ROS levels.

## 5 Conclusion

**Figure 5:**
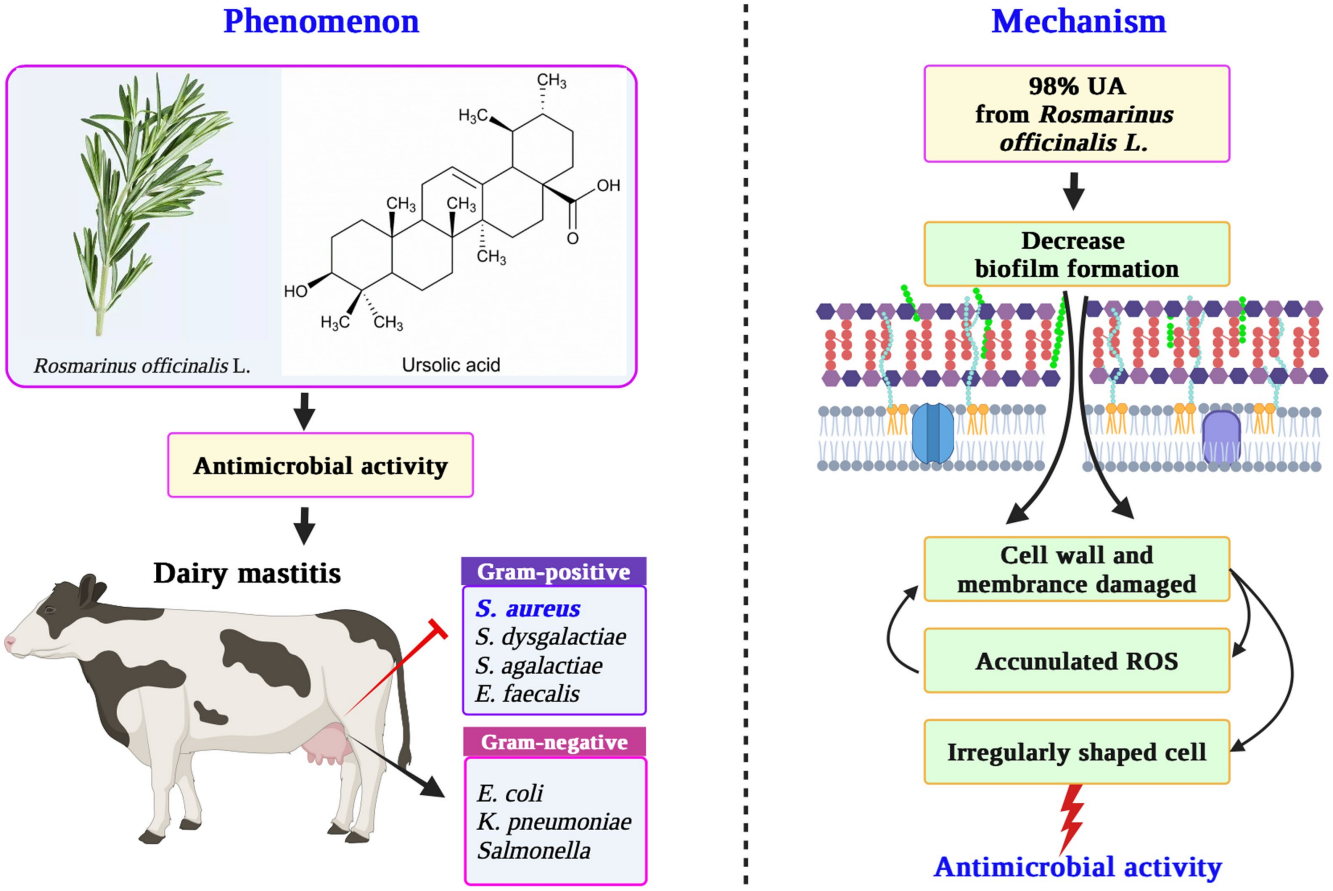
The mechanism for the antibacterial activity of UA from *Rosmarinus officinalis* L. against *S. aureus*. UA, ursolic acid; ROS, reactive oxygen species.

In summary, these findings highlight that 98% UA from *Rosmarinus officinalis* L. exhibits robust antibacterial effects against Gram-positive bacteria without any corresponding impact on Gram-negative bacteria. Clinical *S. aureus* isolates associated with bovine mastitis, when treated with 98% UA at 78 μg/mL, exhibited irregularly shaped cells and the dissolution of cell wall and membrane layers, with the pronounced inhibition of intracellular protein synthesis and high levels of ROS production (Figure 5). Based on these data, UA represents a promising alternative to traditional antibiotics that can be used alone or in combination with extant antibiotic drugs in an effort to treat *S. aureus* infections.

## Data availability statement

The datasets presented in this study can be found in online repositories. The names of the repository/repositories and accession number(s) can be found below: the NGS sequences for the *S. aureus* S4, *S. aureus* S5, and *S. aureus* S6 isolates were submitted to the NCBI database (BioSample accession number SAMN38649273, SAMN38673743 and SAMN38673744).

## Author contributions

GL: Writing – original draft, Writing – review & editing. PQ: Data curation, Project administration, Writing – original draft. XC: Investigation, Supervision, Writing – review & editing. LW: Investigation, Resources, Writing – review & editing. WZ: Software, Writing – review & editing. WG: Funding acquisition, Supervision, Writing – review & editing.
